# Short-Term Cryopreservation Preserved the Function of MSCs from Bone Marrow Aspirate Concentrate

**DOI:** 10.3390/cells14191569

**Published:** 2025-10-09

**Authors:** Jacob Singer, Haruki Nishimura, Zuokui Xiao, Xueqin Gao, Noah Knezic, Laura Chubb, Jonathan E. Layne, Ping Guo, Aiping Lu, Johnny Huard

**Affiliations:** 1 Linda and Mitch Hart Center for Regenerative and Personalized Medicine, Steadman Philippon Research Institute, Vail, CO 81657, USA; jsinger@sprivail.org (J.S.); hnishimura@sprivail.org (H.N.); zxiao@sprivail.org (Z.X.); xgao@sprivail.org (X.G.); nknezic@sprivail.org (N.K.); jlayne@sprivail.org (J.E.L.); pguo@sprivail.org (P.G.); 2 Department of Clinical Science, Colorado State University, Fort Collins, CO 80525, USA; laura.chubb@colostate.edu

**Keywords:** fresh and frozen bone marrow aspirate concentrate (BMAC), mesenchymal stem cell (MSC), chondrogenic differentiation, cartilage repair, osteoarthritis (OA)

## Abstract

**Highlights:**

**What are the main findings?**

- MSC proliferation and multilineage differentiation were preserved after freezing BMAC at −80 °C for 4 weeks.
- Both fresh and frozen BMAC equally improved histological cartilage scores compared with PBS control in an OA rat model.

**What is the implication of the main finding?**

- Frozen BMAC retains functional equivalence to fresh BMAC for cartilage repair.
- A single bone marrow harvest with storage for multiple injections may reduce patient burden and expand clinical utility.

**Abstract:**

Bone marrow aspirate concentrate (BMAC) is increasingly recognized as a valuable orthobiologic, offering promising outcomes in reducing inflammation, alleviating pain for patients with osteoarthritis (OA) and various musculoskeletal conditions. However, BMAC contains a very low percentage of mesenchymal stem cells (MSCs), and multiple injections are often required with multiple harvests, which can lead to scarring at the extraction site and patient discomfort. This study aimed to determine whether freezing BMAC affects the function of MSCs in vitro and their capacity to repair articular cartilage in vivo using an OA rat model. BMAC was obtained from patients undergoing BMAC treatment. The in vitro results showed that the proliferation and multilineage differentiation of MSCs remained similar after being frozen for 4 weeks at −80 °C. In vivo, both fresh and frozen BMAC demonstrated significantly improved ICRS histology score of tibial plateau cartilage compared to the PBS control. No significant difference was found between fresh and frozen BMAC treatment groups. Our results suggest that the freezing process does not negatively affect the function of MSCs from BMAC for cartilage repair. These findings support the potential future applications of a single harvest with BMAC storage for multiple injections, thereby enhancing the tissue repair capabilities of BMAC.

## 1. Introduction

Musculoskeletal injuries and disease, including osteoarthritis (OA), are the most disabling and costly conditions suffered by Americans [1,2]. In a 2019 report, musculoskeletal conditions affected approximately 127.4 million people (more than a third of the U.S. population); they were the main driver of health-care spending, with an estimated direct annual cost of $380.9 billion [3]. Stem cell therapies are rapidly gaining popularity for treating OA and many other orthopedic disorders [4]. Bone marrow aspirate concentrate (BMAC) is a widely acceptable and clinically translatable orthobiologic that is harvested using minimally invasive methods. It is commonly used to treat musculoskeletal injuries and recently has shown promise in improving clinical outcomes in OA, by reducing pain in several clinical trials [5,6,7,8]. BMAC is obtained by processing bone marrow aspirate (BMA) from patients, concentrating it into an injectable form by reducing the presence of erythrocytes while enriching mononuclear cells and mesenchymal stem cells (MSCs) [9], and then injecting it into the injured area to promote healing and regeneration. BMAC therapy represents a significant advancement in the field of regenerative medicine, and it can be used for various conditions, including OA, tendon and ligament injuries, and chronic wounds [10,11]. BMAC is particularly notable for being an autologous treatment, derived from the patient’s bone marrow extracted typically through the iliac crest, and can be acquired through a minimally invasive procedure at a competitive cost compared to other stem cell therapies. Despite increasing clinical use, standardized harvest, processing, and cryopreservation protocols for BMAC are lacking, and comparative data on fresh versus frozen BMAC are limited.

Some components of BMAC play different therapeutic roles for tissue repair, including MSCs, hematopoietic stem cells, growth factors, cytokines and chemokines, platelets and exosomes. MSCs derived from bone marrow and concentrated into BMAC play a significant role in tissue regeneration [12]. MSCs are multipotent stem cells that can differentiate into various tissue types, including bone, cartilage, and skeletal muscle. They also have immune-modulating and anti-inflammatory properties, which are beneficial for the healing process [12,13]. However, BMAC has a very low percentage of functional MSCs, as low as 0.001% [14]. To maintain pain reduction for OA patients and improve tissue healing, multiple injections are often required. Without a widely accepted stem cell banking program, multiple harvests are also required, which can lead to scarring at the extraction site, as well as pain and discomfort for the patients. Cryopreservation represents an efficient method to preserve MSCs for long-term storage and achieve the large numbers of MSCs required for clinical applications, including cell-based therapies and regenerative medicine [15]. It is a challenge to preserve the functional properties of MSCs, including immunomodulatory properties and multilineage differentiation capacity after cryopreservation [16].

With the growing application of BMAC for musculoskeletal injury, there is an increasing need for standardized harvest, preparation protocols, consistent cryopreservation methods, and regulatory approval. It is essential to establish a BMAC harvesting, banking, and delivery system that allows for multiple injections from a single bone marrow harvest without the cell expansion or extensive manipulation. The goal of this study is to determine whether freezing BMAC affects the function of MSCs in vitro and their capacity to repair articular cartilage in vivo, using an OA rat model. The result will provide evidence on whether a singular harvest with storage of BMAC for multiple injections is feasible to enhance the tissue repair capacities of BMAC. We hypothesized that MSC function in vitro and cartilage repair in vivo can be preserved after cryopreservation under specific conditions.

## 2. Materials and Methods

### 2.1. Patient Recruitment and BMAC Collection

Study participants were recruited between March 2023 and May 2025 (n = 15, 9 male and 6 female, age between 32 and 55 years) at the Steadman Clinic (TSC) in Vail, Colorado (IRB#2020-050). BMAC was obtained through TSC and processed by ProofPoint Biologics (PPB) using a similar protocol as previously described [9,17]. PPB is a CLIA- and COLA-accredited clinical laboratory and service line of TSC. PPB produces peripheral blood derivatives, platelet-rich plasma (PRP), and bone marrow concentrate (BMAC) for homologous treatment of musculoskeletal disorders. Residual BMAC and plasma were then obtained in Steadman Philippon Research Institute (SPRI) to be further analyzed. Three additional BMA samples were purchased from STEMCELL Technologies (Cambridge, MA, USA. n = 3, male, aged between 29 and 52 years). One BMAC sample was obtained from Dr. Jeffrey Donner (Colorado Spine Institute, IRB #20230413, 51-year-old, female) for the in vivo experiment.

### 2.2. Preparation of Frozen BMAC and Plasma

Fresh BMAC was obtained from PPB and aliquoted appropriately for experiments. Half of the fresh BMAC was centrifuged at 1500× *g* for 10 min; the buffy coat was collected and then resuspended in 10% dimethyl sulfoxide (DMSO) and 90% autologous plasma, frozen using a freezing container, and stored in a −80 °C freezer. The fresh plasma aliquots from BMAC were analyzed immediately as part of the fresh cohort, and half were stored for 1 month at −80 °C for analysis.

### 2.3. Mononuclear Cell Isolation from Fresh and Frozen BMAC and Platelets Measurement

BMAC was centrifuged at 1500× *g* for 10 min. The plasma was collected and saved for freezing mononuclear cells. The remaining sample was diluted 1:1 with dilution media (PBS + 2% FBS). A Ficoll Gradient (Cytiva, Marlborough, MA, USA. Cat# 17144002) was prepared using 15 mL of Ficoll Gradient solution in a 50 mL tube, and the sample was carefully added to maintain the density barrier. The 50 mL tube was spun at 400× *g* for 40 min. Following the spin, the Buffy Coat layer was carefully extracted and transferred to a new tube, then washed twice with 15 mL of dilution media and spun at 300× *g* for 8 min. Cells were counted. Cells for fresh BMAC experiments were cultured for various in vitro experiments. Cells for frozen BMAC experiments were resuspended in 10% DMSO and 90% autologous plasma at a 1 million cells/mL density. Samples were cooled in a Mr. Frosty passive controlled-rate container (~−1 °C/min) and stored for four weeks in a −80 °C freezer. Four weeks later, the frozen cells were rapidly thawed in a 37 °C water bath, followed by dilution with pre-warmed culture medium. They were then spun at 300× *g* for 5 min to remove DMSO and then cultured under optimal conditions. Mononuclear cells from fresh and frozen BMAC were plated in growth media (alpha minimum essential medium eagle, aMEM with 20% FBS, 1% Penicillin/Streptomycin, and 10 ng/mL FGF-2) in tissue culture-treated plates. Cells were grown out to passage 2 (P2) to purify MSCs from other mononuclear cells and obtain a cell count as high as required for the differentiation assay. The number of platelets in the fresh and frozen BMAC was measured via a Ruby automated hematology analyzer (Abbott, Lake County, IL, USA) in its standard CBC mode, following the manufacturer’s instructions.

### 2.4. Colony Forming Units-Fibroblast (CFU-f) Assay

Mononuclear cells from fresh and frozen BMAC were plated at 300,000 cells/well in a six-well plate with three wells per condition per donor. Cells were cultivated for 14 days in aMEM medium supplemented with 20% FBS, 1% Penicillin/Streptomycin, and 10 ng/mL FGF-2, and then fixed with 4% paraformaldehyde (PFA) (Alfa Aeser, Ward Hill, MA, USA. Cat #J19943K2) and dyed for 30 min with 1% crystal violet solution (Sigma-Aldrich, Burlington, MA, USA. Cat# V5265-500ML). Colonies were counted manually on day 14 using a pre-specified criterion of over 100 cells to determine the number of colonies per 300,000 seeded cells. Colonies were counted only when a distinct clonal center was evident, and confluent clusters were scored as a single colony unless visually separable clonal centers were divided by a clear boundary. Ambiguous clusters were counted as one. Technical replicates were averaged to a single donor value for analysis.

### 2.5. Multiplex Analysis to Detect Soluble Factors in the Plasma from BMAC

The BMAC plasma from three different donors was selected for multiplex analysis. The fresh and frozen plasma samples were run on two different Multiplex kits, i.e., the 11-plex cytokine/Chemokine (Millipore, Burlington, MA, USA. HCYTOMAG-60K) and Transforming Growth Factor-β (TGF-β 1, 2, 3) (Millipore, TGFBMAG-03) kits. The data was analyzed via MAGPIX. Predefined Luminex QC criteria were applied and excluded analyte-specific values when bead recovery was lost (i.e., the instrument recorded insufficient beads) and duplicate precision was unacceptable, yielding a sample number of 3–4, depending on analyte.

### 2.6. In Vitro Adipogenic Differentiation Assay and Oil Red O Staining

MSCs at Passage 2 were then plated at a density of 35,000 cells/well in a 24-well plate for adipogenic differentiation according to the manufacturer’s protocols provided by Lonza (Walkersville, MD, USA. PT-3004). Eighteen days after adipogenic differentiation, Oil Red O Staining was performed using the method previously described [18] Briefly, the cells were fixed with cold 4% PFA (Alfa Aeser, J19943K2) for 15 min and washed twice with water. The cells were then incubated with 60% isopropanol for 5 min, followed by an incubation with 500 uL of Oil Red O working solution for 15 min at room temperature. The working solution was prepared by mixing three parts of Oil Red O stock (made from 60 mg of Oil Red O (Sigma Aldrich, MAK194C) in 20 mL of 100% isopropanol) with two parts of water. The working solution was thoroughly mixed and filtered through Whatman No. 1 filter paper before use. Following incubation, the cells were washed 3 times with distilled water and then imaged using the Nikon Eclipse Ni-U microscope (Nikon, Melville, NY, USA). Following imaging, the dye was collected by adding 500 mL of isopropanol per well and incubating on a rocker for 15 min. Next, 200 mL of dye from each well was collected and put into a 96-well plate, and the optical density was measured at 492 nm.

### 2.7. In Vitro Osteogenic Differentiation Assay and Alizarin Red Staining

MSCs at P2 were grown in Lonza Osteogenic differentiation media (Cat# PT-3002) supplemented with BMP-2 (10 ng/mL) for 14 days. Alizarin-Red staining was performed, and bright-field images were captured using a Nikon Inverted microscope. To quantify the alizarin red staining, the dye was solubilized by incubating the cells for 15 min using a 10% (*w*/*v*) cetylpyridinium chloride (CPC) (Sigma Aldrich, C0732) in PBS with moderate shaking. The final solutions were added to a 96-well plate, and the optical density was measured at 570 nm [18].

### 2.8. In Vitro 3D Pellet Culture for Chondrogenic Differentiation

MSCs from fresh and frozen BMAC were obtained from 3 patients to compare chondrogenic potential through 3D pellet culture as previously described [19]. Cells were grown to Passage 2 to purify MSCs, which were then distributed 5 × 10^5^ cells/tube into 6 × 15 mL conical tubes and centrifuged at 200× *g* for 5 min to form a pellet. Cell pellets were cultured in chondrogenic media supplemented with TGF-β 1 (10 ng/mL) and bone Morphogenic Protein 4 (BMP-4) (50 ng/mL). Pellets were subjected to medium change every 4–5 days, and pellets were harvested 30 days after culture initiation. For histological analysis, the chondrogenic pellets were fixed in 4% neutral buffered formaldehyde for 1 h, and their diameters were measured using ImageJ 1.54g with a ruler as a reference expressed in micrometers (µm). Pellets were then embedded in NEG freezing medium. Cryosections were cut at an 8-μm thickness for histology and collagen II staining.

### 2.9. Immunohistochemistry

Paraffin slides of all groups were deparaffinized and rehydrated. Antigen retrieval was performed using 2% hyaluronidase (Sigma-Aldrich/Millipore, H3506) in PBS (pH = 7.4) at room temperature for 30 min. Slides were then washed with PBS and incubated with 5% donkey serum in PBS at room temperature for 1 h. The sections were then incubated with mouse anti-collagen II (Col2) (Invitrogen/Fisher, MA1-37493) at a 1:100 dilution overnight at 4 °C. On the second day, the slides were washed with PBS. The slides were then immersed in 0.5% hydrogen peroxide in PBS for 30 min to inactivate endogenous hydrogen peroxidase. After an additional wash with PBS, slides were incubated with biotinylated horse anti-mouse secondary antibody (Vector Laboratories, Burlingame, CA, USA. BA2000) for 2 h at room temperature. The slides were subsequently incubated using the Elite ABC kit (Vector Laboratories, PK-6100) after being washed in PBS. DAB color reaction was performed using the DAB Kit (Vector Laboratories, SK-4100) for 6 min. Slides were rinsed with tap water thoroughly and nuclei counterstaining was performed using Hematoxylin QS (Vector Laboratories, H-3404-100) for 20 s. Subsequently, the slides were rinsed in running tap water for 10 min before dehydration using a gradient of ethanol and xylene and then cover-slipped with a xylene-based Cytoseal mount medium (Fisher Scientific, Waltham, MA, USA).

### 2.10. q-PCR Analysis

After 30 days of culture, three pellets from each population of fresh and frozen BMAC-derived MSCs were flash-frozen in liquid nitrogen and stored at −80 °C for quantitative PCR (q-PCR) analysis. RNA was extracted from three fresh and frozen chondrogenic pellets using a Qiagen RNA isolation kit (Qiagen, Germantown, MD, USA. Cat# 74104), converted into cDNA, and subsequently analyzed for qPCR Col 2a1, Col 10a1, and PRG4 (Proteoglycan 4) gene expression using Taqman Probes (Themo Fisher, Waltham, MA, USA. Cat# 4331182, Assay ID, Hs00264051, Hs00166657, Hs00981633). The markers of MSCs isolated from fresh and frozen BMAC without culturing have also been tested by q-PCR. For the in vivo study, articular cartilage from both the medial tibial plateau and the femoral condyle of rats was collected from the injured knees of five rats in each group (PBS, fresh BMAC, frozen BMAC), providing five biological replicates per group. Each biological sample was analyzed in triplicate (technical replicates). Technical replicates were used to ensure measurement reliability, while the independent samples from different animals represented biological variation. The cartilage was homogenized in Trizol and RNA extraction was performed using Trizol (Invitrogen, Themo Fisher), cDNA synthesis and q-PCR were subsequently performed using Q-Quant reagents following the manufacturer’s protocol. Primers and Taqman probe for each gene were purchased from Thermo Fisher Scientific, and their sequence can be found in Table 1. Gene expression levels were analyzed using the ΔΔCt method. GAPDH was used as the endogenous reference gene, and the PBS group served as the calibrator, which was set to 1.0 by definition.

### 2.11. Surgical Induction of Osteoarthritis and Human BMAC Treatment

This study was approved by IACUC of Colorado State University (Protocol# 1322). First, 12-week-old female RNU nude rats (purchased from Charles River Laboratories, Wilmington, MA, USA) were divided into three groups (N = 10/group), PBS control, fresh-BMAC and frozen-BMAC. All rats were subjected to destabilization of the medial meniscus (DMM) surgery by following the previously published method [20]. BMAC was split into fresh and frozen portions. For the fresh-BMAC group, 100 uL of BMAC was injected into the injured knee when OA was developed at 4 weeks post-surgery under isoflurane anesthesia. BMAC was frozen by separating cells and plasma. Briefly, BMAC was spun down, plasma was aspirated in separate tubes and frozen directly. Cells were frozen in a freezing medium containing 10% DMSO and 90% autologous plasma at a density of 1 million cells/mL and placed into a freezing container. They were stored in a −80 °C freezer for 4 weeks. At the time of injection, cells were thawed in a 37 °C water bath, followed by dilution with human plasma, and spun at 300× *g* for 5 min to remove DMSO. The viable nucleated cells were quantified, and they were then combined with frozen plasma for injection. The number of living cells was adjusted based on cell viability (53% ± 27%) so that the viable cell dose per 100 μL matched that of the fresh-BMAC group. A quantity of 100 μL of thawed BMAC was injected into the injured knee under isoflurane anesthesia. Rats were sacrificed 8 weeks after injection and rat knees were harvested, and the knees diameter was measured using digital calipers before dissection. Then, five rats from each group were dissected to separate the femur and tibia for gross observation and imaging. Macroscopic evaluation was performed using the ICRS score, as reported previously [21].

### 2.12. Micro-CT Scanning

Another five rats from each group were fixed in 10% neutral buffered formalin for 5 days and subjected to micro-CT scanning of the proximal tibia. MicroCT was performed for proximal tibia ex vivo using a Scanco VivaCT 80 (Scanco Medical AG, Brüttisellen, Switzerland. CH-8306 Bruettiselllen) with the following settings: 70 kVp, 114 µA, 58 W, 10.0 µm voxel size, 200 ms integration time, and 31.9 mm field of view for a 10 µm resolution. Three-dimensional images were reconstructed and analyzed using Scanco Medical Software (v 7.1.2.). Analysis was performed on 70 slices around the center of the epiphysis and divided into the subchondral plate and trabecular part for both the medial and lateral tibial plateaus. A 1 cm × 0.5 cm size region was analyzed in each for cortical thickness (Ct.Th). Trabecular bone was analyzed for bone volume (BV), tissue thickness (TV), thickness (Tb.Th.), spacing (Tb.Sp.), and connectivity density (CD). All analyses were performed using the trabecular bone analysis script in the Scanco Software (v4.2.).

### 2.13. Histology

After Micro-CT scanning, the entire knee of each rat was decalcified using commercialized formic acid for one month. Tissues were then processed using the Heide automated tissue processor with gradient alcohol, cleared with xylene, and immersed in 3 steps of paraffin 9 (Thermofisher Scientific) according to a previously described protocol [20,22,23]. Paraffin sections 5 μm thick were cut using a microtome.

Alcian blue and Safranin O staining was performed using the IHC world protocol as previously described [24,25,26].

### 2.14. Statistical Analysis

All continuous data were analyzed using GraphPad Prism 10 software. Two-group comparisons used a *t*-test when assumptions were met or Mann–Whitney when not; multi-group comparisons used ANOVA with Tukey’s post hoc when assumptions were met or Kruskal–Wallis with Dunn’s post hoc otherwise. *p* < 0.05 was considered statistically significant.

## 3. Results

### 3.1. Effect of Freezing on Platelets in the BMAC

**Figure 1:**
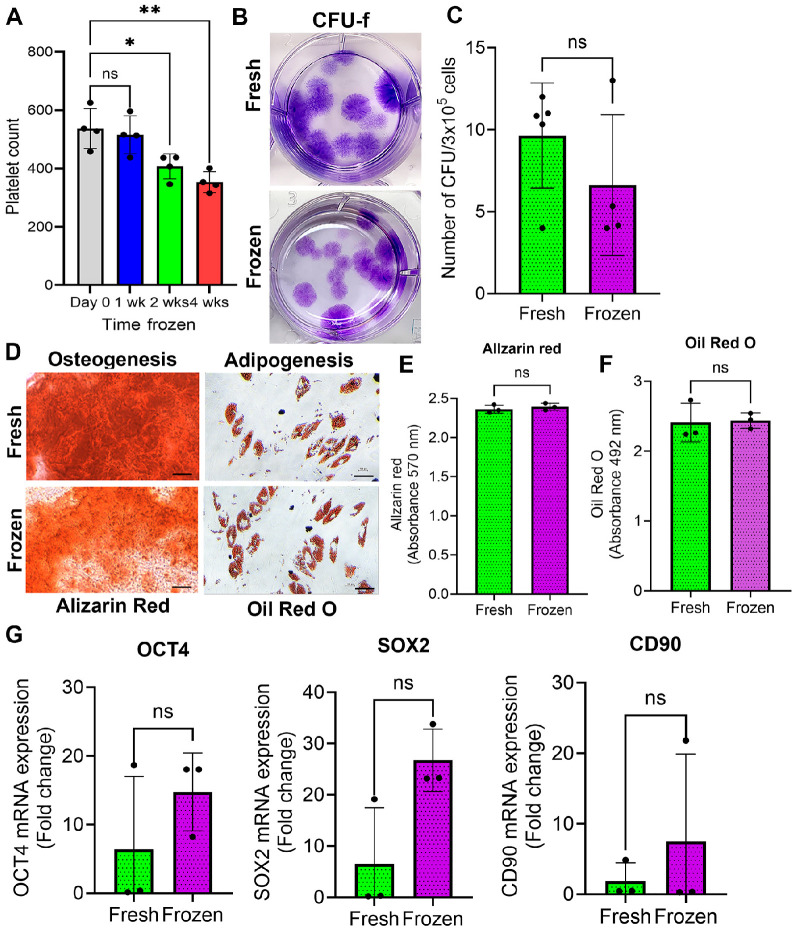
**In vitro characterization of MSCs isolated from fresh and frozen BMAC.** (**A**). Platelet counts and quantification (n = 4). (**B**). Representative images of CFU-f from fresh and frozen BMAC. (**C**). Quantification of CFU-f from fresh (n = 5) and frozen (n = 4) BMAC. The cells were seeded at 300,000 cells/well in a six-well plate for each sample. Colonies were counted manually on day 14 using a pre-specified criterion of over 100 cells to determine the number of colonies per well (one frozen donor sample exceeded the 4-week storage window, and it was excluded). The same criteria were applied to fresh and frozen samples. (**D**). Representative images of Alizarin red and Oil red O staining. (**E**). Quantification of Alizarin red staining (n = 3). Quantification is derived from 570 nm absorbance. (**F**). Quantification of Oil red O staining (n = 3). (**G**). q-PCR analysis of MSC markers. Error bars indicate ‘mean + SD’, ns = not significant, * *p* < 0.05, ** *p* < 0.01. In panel (**D**), all scale bars are set to 100 µm.

BMAC is a bone marrow product that contains platelets, which play a crucial role in tissue healing and regenerative processes within BMAC. The number of platelets in the fresh and frozen BMAC were measured via a Ruby Hematology analyzer. We found a significant decrease in the platelet counts after BMAC was frozen for 2 and 4 weeks (Figure 1A), indicating that long-term freezing of BMAC will affect the number of platelets in BMAC.

### 3.2. Effect of Freezing on CFU-f Formation

The CFU-f assay is a cell culture technique to determine the frequency of plastic adherent of MSCs contained in the BMAC. Each colony is generated from a single MSC, and the number of colonies observed is representative of the number of MSC as a fraction of the total cells plated [27,28]. To determine if cryopreservation affects CFU-f frequency, we performed a CFU-f assay on the mononuclear cells from fresh and frozen BMAC without culturing. By counting the number of colonies, we observed considerable variation in both fresh and frozen BMAC samples; however, no significant difference was found between fresh and frozen BMAC in terms of the frequency of CFU-f (Figure 1B,C), although cryopreserved BMAC yielded viable nucleated cells in 53% ± 27% upon thawing. These results indicate that cryopreservation did not affect the number of MSCs and their proliferation capacity.

### 3.3. Effect of Freezing on Adipogenic and Osteogenic Differentiation of MSCs and Markers of MSC

MSCs are defined as multipotent stem cells that can differentiate into multilineages [29]. Therefore, to determine whether cryopreservation of BMACs affects the ability of MSCs to differentiate toward different lineages, the MSCs were isolated from fresh and frozen BMAC, and cultured to P2 to obtain a sufficient number of cells. The adipogenic and osteogenic lineage differentiation potential of MSCs from fresh and frozen BMACs was compared. Alizarin red staining showed the formation of calcium oxalates on the differentiated MSCs, and no difference was found between fresh and frozen BMAC by quantifying the Alizarin red staining (Figure 1D,E). Oil Red O-stained intracellular lipid droplets are the standard method to confirm the adipogenic differentiation of MSCs, and no difference was found between fresh and frozen BMAC by quantifying the Oil Red O staining (Figure 1D,F). The result revealed that the MSCs from both fresh and frozen BMAC were able to undergo osteogenesis and adipogenesis with no significant difference between fresh and frozen groups, indicating that the function of MSCs remains similar after being frozen for 4 weeks. MSC surface markers have been used to identify and compare various stem cell populations. A reduction in MSC differentiation capacity was associated with a decreased level of stem cell surface markers [30]. To determine if cryopreservation affects the markers of MSC, we examined some MSC markers by q-PCR, including CD90, OCT4, and Sox2 in fresh and frozen BMAC samples. The result showed no significant difference in the expression of those MSC markers expression between fresh and frozen BMAC (Figure 1G).

### 3.4. Effects of Freezing on Chondrogenic Differentiation of MSCs

**Figure 2:**
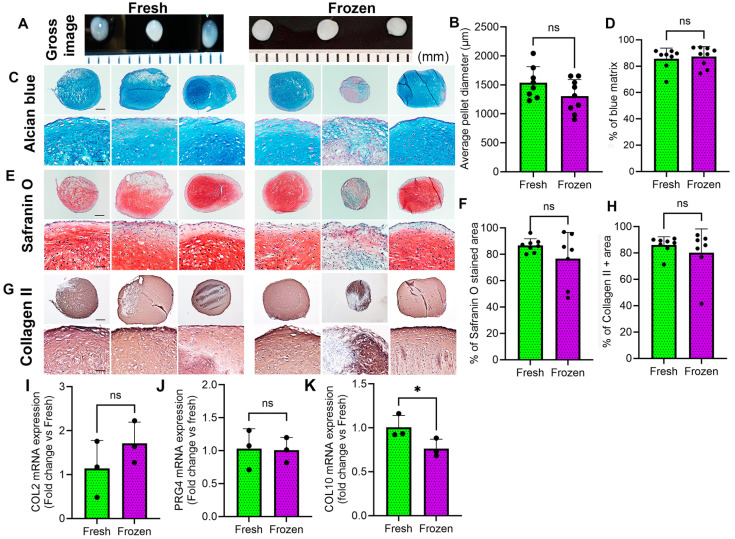
**In vitro chondrogenic differentiation of MSCs from fresh and frozen BMAC.** (**A**,**B**). Gross images of chondrogenic pellets of each group. (**C**). Alcian blue staining of chondrogenic pellets. Hyaluronic acid and acid mucin stained blue. (**D**). Quantification of blue matrix percentage. (**E**). Safranin O staining for GAG. GAG stained orange-red color. (**F**). Quantification of orange-red stained GAG percentage. (**G**). Immunohistochemistry of Col2. Col2 stained violet red. (**H**). Quantification of the percentage of Col2A. (**I**–**K**). q-PCR analysis of COL2, PRG4, and COL10 mRNA of pellets derived from fresh and frozen MSCs. Error bars indicate ‘mean + SD’, n = 3. Each sample was run in triplicate. One fresh pellet was lost due to technical issues. ns = not significant, *p* > 0.05, * *p* < 0.05. In panels (**C**,**E**,**G**), the top images have all scale bars set to 250 µm, while the bottom image has all scale bars set to 50 µm.

In osteoarthritis (OA), chondrogenesis is often impaired, leading to cartilage degradation [31]. To determine if the chondrogenic potential of MSCs can be preserved after freezing, chondrogenic differentiation assays and q-PCR analysis were performed to assess the chondrogenic-associated gene expression. MSCs from fresh and frozen BMAC were obtained from three patients to compare chondrogenic potential through 3D pellet culture. Our results showed no significant difference in pellet diameter between fresh and frozen MSCs/BMAC (Figure 2A,B). Alcian blue staining yielded nearly complete blue staining for pellets from both fresh and frozen MSCs/BMAC. There were no significant differences in the quantification of the percentages of Alcian blue-positive matrix between the two groups (Figure 2C,D). Safranin O staining resulted in nearly complete orange-red staining for pellets from both fresh and frozen MSCs. There were no significant differences in the quantification of the percentages of orange-red matrix between the two groups (Figure 2E,F). Additionally, we performed immunochemical staining of COL2. We found strong COL2 staining with a violet-red color in almost all the pellets of both groups. We found no statistical difference between fresh and frozen groups regarding the percentage of COL2 matrix (Figure 2G,H). Furthermore, q-PCR analysis revealed no significant difference in COL2 and PRG4 expression between fresh and frozen chondrogenic pellets (Figure 2I,J). However, COL10 expression was significantly decreased for frozen pellets compared to fresh BMAC-derived MSCs (Figure 2K). The results indicate that the chondrogenic potential of MSCs remains similar after their being frozen for one month.

### 3.5. Effect of Freezing on Soluble Factors in the Plasma from BMAC

**Figure 3:**
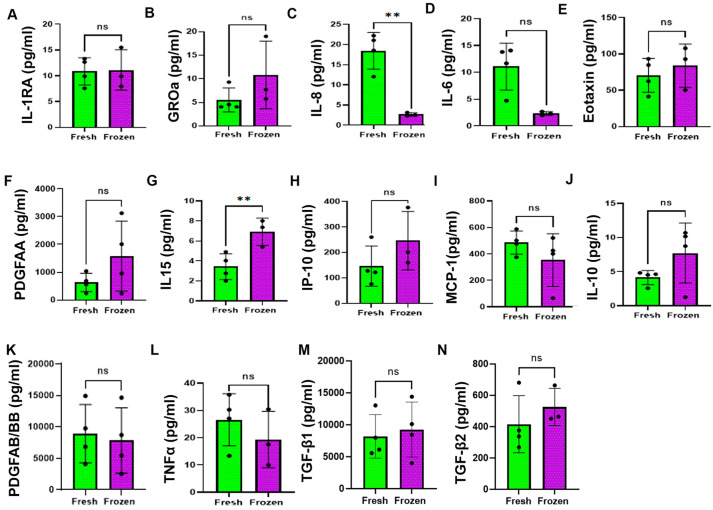
**Comparison of growth factor concentration between fresh and frozen plasma from BMAC**. (**A**). IL-1RA. (**B**). GROa. (**C**). IL-8. (**D**). IL-6. (**E**). Eotaxin. (**F**). PDGF-AA. (**G**). IL-15. (**H**). IP-10. (**I**). MCP-1. (**J**). IL-10. (**K**). PDGFAB/BB. (**L**). TNFα. (**M**). TGF-β1. (**N**). TGF-β2. Error bars indicate ‘mean + SD’, n = 3/4 (some samples were excluded due to low bead recovery as we applied predefined Luminex QC analyte specific values). ns = not significant, *p* > 0.05, ** *p* < 0.01.

In BMAC therapy, soluble factors in the plasma from BMAC play a crucial role in facilitating healing and potentially enhancing the regenerative capacity of stem cells within the BMAC [32,33]. The growth factors in the plasma from the BMAC can further support the healing process by stimulating the proliferation and differentiation of stem cells. To investigate whether the freezing process affects the concentration of growth factors in the plasma of BMAC, we have detected cytokines and chemokines in the plasma from BMAC using multiplex assays, and we compared the differences between fresh and frozen BMAC. We observed that most of the factors in frozen plasma remain at the same concentration as fresh plasma, except a decreased IL-8 and increased IL-15 level in frozen BMAC plasma compared to fresh BMAC plasma (Figure 3A–N). This result suggested that freezing BMAC for 4 weeks did not change the concentration of the growth factor in the plasma of BMAC except, for IL-8 and IL-15.

### 3.6. No Significant Difference Between Fresh and Frozen in the AC Regenerative Potential of BMAC

**Figure 4:**
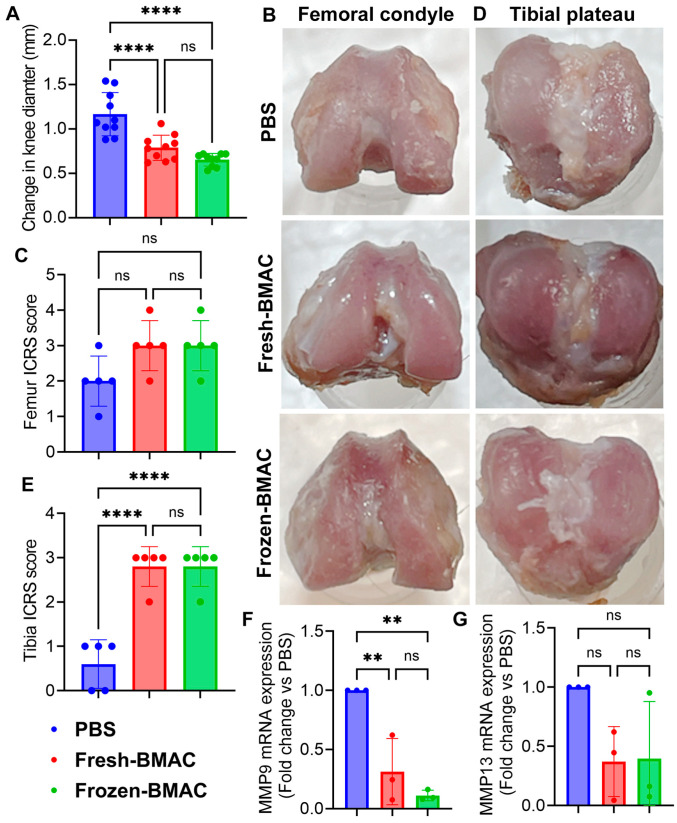
**The macroscopic evaluation of knees after fresh and frozen BMAC treatments.** (**A**). Quantification of knee diameter measurement (n = 10). (**B**). Gross images of the femoral condyle. (**C**). Femur ICRS score (n = 5). (**D**). Gross pictures of the tibial plateau. (**E**). Tibia ICRS score (n = 5). (**F**,**G**). q-PCR analysis of MMP9 and MMP13 mRNA of dissected cartilage derived from fresh and frozen BMAC treatment. (n = 3). Error bars indicate ‘mean + SD’, ns = not significant, *p* > 0.05, ** *p* < 0.01, **** *p* < 0.0001.

To further determine whether the frozen BMAC can maintain its regenerative capacity in vivo, we created a DMM OA model in rats. We treated them with both fresh and frozen BMAC. Eight weeks after BMAC treatment, the rats were euthanized. We first measured the knee diameter and found that both fresh and frozen BMAC treatment decreased the knee diameter of nude rats compared to the PBS-treated group (Figure 4A). There was a trend to improve the femoral condyle macroscopic score for both fresh-BMAC and frozen BMAC treatment, but no statistically significant difference when compared to the PBS group (Figure 4B,C). However, both fresh and frozen BMAC significantly improved the macroscopic score of the tibial plateau cartilage compared to the PBS group; however, no significant difference was found between fresh-BMAC and frozen-BMAC (Figure 4D,E). Matrix metalloproteinases 9 (MMP9) and 13 (MMP13) are crucial enzymes involved in cartilage degradation, especially in OA [34]. We isolated articular cartilage from the knees in different groups and performed q-PCR to check the MMP9 and MMP13 expressions. The results showed that both fresh and frozen-BMAC treatment groups had significantly decreased MMP9 mRNA expression in the injured articular cartilage of the knee (Figure 4F). Additionally, both fresh and frozen-BMAC groups also showed a trend of reducing MMP13 expression (Figure 4G). No difference was found between fresh and frozen BMAC treatment groups.

### 3.7. Neither Fresh nor Frozen BMAC Treatment Significantly Affected Epiphysis and Subchondral Bone

**Figure 5:**
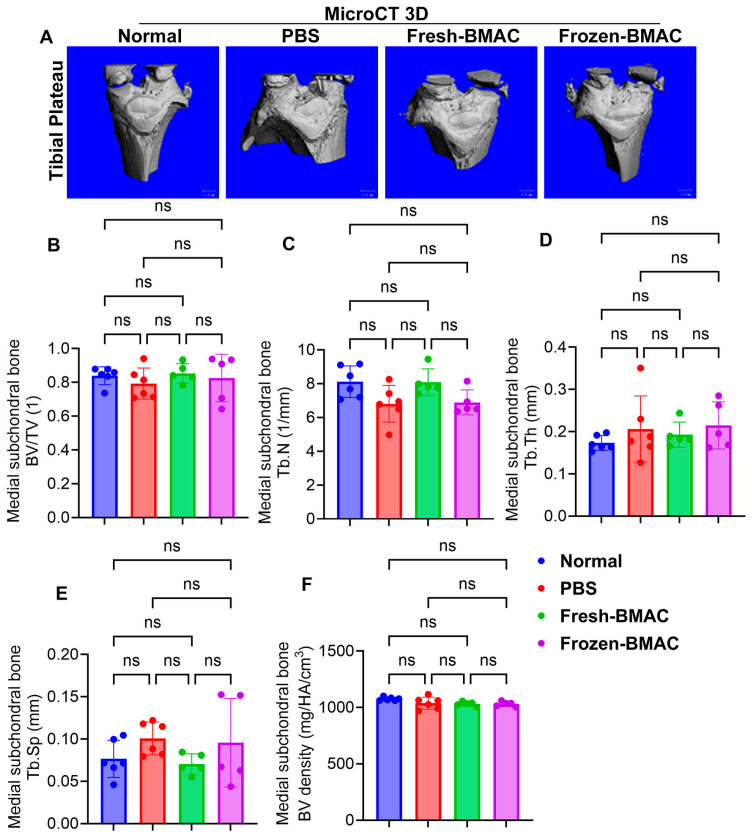
**Micro-CT 3D and quantification of medial tibial plateau subchondral bone.** (**A**). Micro-CT 3D images of tibial plateau. (**B**–**F**), Quantification of BV/TV, Tb.N, Tb.Th, Tb.Sp, and BV density of the medial tibia plateau subchondral bone. Error bars indicate ‘mean + SD’. ns = not significant, *p* > 0.05.

As we know, in OA, the epiphysis and subchondral bone are also affected by progressive cartilage damage. We next performed micro-CT scanning and quantified the microarchitecture of the epiphysis and subchondral bone on both the lateral and medial sides of the tibial plateau. We found no significant difference in all bone microarchitecture parameters of lateral epiphysis, subchondral bone, and medial epiphysis trabecular bone for both fresh and frozen BMAC treatment (Figure 5A–E). However, the PBS group showed a trend of decreasing trabecular number in the medial subchondral bone (Figure 5A–F).

### 3.8. Both Fresh and Frozen BMAC Treatment Improved the Histology Score of Tibial Plateau Cartilage

**Figure 6:**
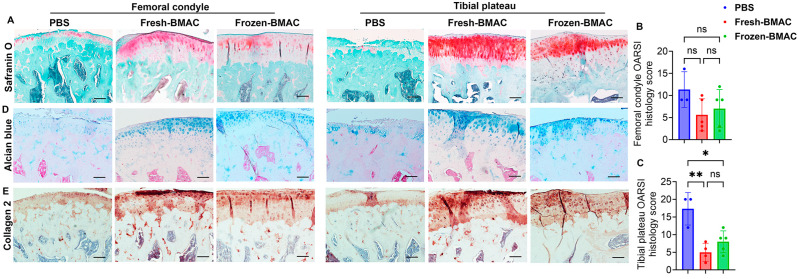
**Histology analysis of the knee joint 8 weeks after BMAC treatment**. (**A**). Safranin O staining of femoral condyle and tibial plateau cartilage. Glycosaminoglycan (GAG) stained orange-red. (**B**). Quantification of femoral condyle cartilage OARSI histology score (n = 5). (**C**). Quantification of tibial plateau cartilage OARSI histology score (n = 5). (**D**). Alcian blue staining: chondrocytes and cartilage matrix are stained blue. (**E**). Immunohistochemistry of COL2. Error bars indicate ‘mean + SD’. Scale bars = 100 µm. ns = not significant, *p* > 0.05, * *p* < 0.05, ** *p* < 0.01.

The result from Safranin O staining, which detects cartilage matrix glycosaminoglycans (GAGs), showed that much of the cartilage area was superficial and there was a loss of mid-zone chondrocytes in the femoral condyle cartilage for the PBS-treated group. In contrast, the fresh and frozen BMAC groups had most of the cartilage repaired with GAG-positive cartilage when compared to the PBS group (Figure 6A). We indeed observed there was more GAG-positive cartilage in fresh BMAC treated group than in the frozen BMAC treated group (Figure 6A). However, OARSI histology score quantification indicated that the fresh and frozen BMAC groups had a lower OARSI score when compared to the PBS group. Still, there was no significant difference between fresh and frozen BMAC treated groups (Figure 6B). The tibial plateau cartilage of the rats showed severe cartilage damage in the PBS group; some areas showed denudation with complete GAG loss. However, both fresh and frozen BMAC groups exhibited strong orange-red staining in most of the cartilage area. Quantification demonstrated that both fresh and frozen BMAC had a significantly lower OARSI histological score compared to the PBS group, but no significant difference was found between the fresh and frozen BMAC treated groups (Figure 6C). Furthermore, we performed alcian blue staining to detect hyaluronic acid and acid mucin, and the result was similar to what was observed in Safranin O staining. In the femoral condyle cartilage, blue staining was weak due to a loss of chondrocytes or cartilage damage. In the tibial plateau cartilage, the blue matrix showed significant loss with some areas of subchondral bone exposed. In contrast, the tibial plateau cartilage was repaired with chondrocytes similar to normal cartilage, although some fibrillation or fissures could still be observed in some areas (Figure 6D). Immunohistochemistry staining of COL2 demonstrated significant loss of COL2 in the PBS group due to a loss of chondrocytes, while strong COL 2 staining was found in both chondrocytes and the extracellular matrix in fresh and frozen-BMAC treated groups (Figure 6E). Importantly, no significant difference was found between fresh and frozen BMAC groups. This result suggested that frozen BMAC treatment has a similar effect on cartilage repair. When compared to fresh BMAC treatment, the regenerative capacity of MSCs has not been significantly affected after being frozen for one month.

## 4. Discussion

In this study, we compared the function of MSCs derived from fresh versus frozen BMAC (4 weeks at −80 °C) in vitro and their efficacy in cartilage repair in vivo. In vitro, osteogenic, adipogenic, and chondrogenic capacities of MSCs were similar between fresh and frozen BMAC; the CFU-f frequency did not differ between those two groups. In vivo, both fresh and frozen BMAC improved macroscopic and histologic scores relative to PBS, with no difference between the two treatment groups. Those results suggested that MSC viability and function were preserved following cryopreservation.

As we know, the MSCs in BMAC play a crucial role in tissue regeneration, reducing inflammation, and managing pain [35]. It is essential to preserve the immunomodulatory properties and multilineage differentiation ability of MSCs, and cryopreservation is currently the only method to maintain MSCs for a considerable period. Additionally, the biosafety evaluation of cryopreserved MSCs is crucial before their clinical application. Although the effect of cryopreservation on the MSC differentiation ability has been evaluated in many studies [33,34,35], a definitive conclusion has not been reached so far. In this current study, we observed that the MSCs from frozen BMAC have similar capacities of osteogenesis, adipogenesis, and chondrogenesis when compared to the MSCs from fresh BMAC in vitro, indicating the functional MSCs in the BMAC were not affected by the cryopreservation used in this study. Since chondrogenesis is most important for BMAC treatment on OA, we also evaluated some chondrogenic associate genes expression including Col2, Col10, and PRG4. Interestingly, Col2 and PRG4 expressions have not been altered after being frozen for 4 weeks, but the expression of the Col10 gene, which is highly expressed in hypertrophic chondrocytes, was significantly decreased in the MSCs from frozen BMAC. The mechanism of how it can influence the outcome of BMAC treatment for AC repair needs to be further investigated. Furthermore, when injected into the knee joint of the osteoarthritis rat model using the same number of live cells, both fresh and frozen BMAC improved the macroscopic score and the histological score of tibial plateau cartilage compared to the PBS control group. Most importantly, there was no significant difference between fresh BMAC and frozen BMAC. Studies have shown that MMP9 and MMP13 are elevated in articular cartilage, synovial membrane, and synovial fluid of OA patients and contribute to cartilage degradation, leading to increased OA severity [36,37,38,39]. We found that both fresh BMAC and frozen BMAC significantly decreased MMP9 expression compared to the PBS control in the injured articular cartilage, and no difference was found between the fresh and frozen BMAC treatment. This indicates that the functional cells in BMAC are not significantly affected by storing at −80 °C for 4 weeks or by the thawing procedure. Our q-PCR results showed that cryopreservation, rather than decreasing the mRNA expression of the MSC markers, including CD90, OCT4, and Sox2, had a trend to increase their expression. Overall, there was no significant difference between these changes, which is likely due to substantial variations in the BMAC samples between patients. Some studies show that cryopreservation has no significant effect on key markers of MSC [40,41,42] that are consistent with our findings.

The CFU-f assay is a method used to assess the number of progenitor cells as well as to measure the MSC proliferation [28,43]. It has been reported that the numbers of CFU-f in fresh and frozen BMAC are strongly correlated and have similar areas and densities [28]. In our study, we performed a CFU-f assay on mononuclear cells isolated from fresh and frozen BMAC and observed no significant difference in the CFU-f frequency between freshly prepared and cryopreserved BMAC samples. We also tested the storage of mononucleated cells from BMAC in a −80 °C freezer for 4 weeks, which may explain the low cell viability of the cryopreserved BMAC. However, it did not affect the CFU-f frequency, indicating that cryopreservation is a viable method for retaining a significant portion of the progenitor cell population in BMAC, even stored at −80 °C. As we know, BMAC contains a variety of cells, including MSCs, hematopoietic progenitor cells, fibroblasts, macrophages, and endothelial precursor cells [44], while MSCs are a small fraction of the total bone marrow cell population (around 0.001%) [14]. These cells are naturally present in bone marrow; it is unknown how they work together in regenerative medicine to promote tissue repair and healing. Therefore, it is key to maintaining the cell viability for stem cell banking that the method of cryopreservation needs to be optimized. A controlled-rate freezing container, −80 °C freezer and the eventual transfer to liquid nitrogen for long-term cryogenic storage are required during the freezing process. Additionally, when mononucleated cells are isolated from frozen BMAC, red blood cell contamination may lead to reduced cell viability. It is essential to maintain high cell viability to prevent dead cells from transmitting to the recipients.

BMAC is a composite biologic, and the freeze–thaw process may affect both the cellular compartment and the soluble/vesicular factors that contribute to their regenerative potential. In the BMAC, the MSCs provide a direct source of cells for repairing various tissues and exert a paracrine effect, delivering growth factors and cytokines to promote healing and modulate the immune system [33]. Although many clinical studies have demonstrated the beneficial effects of BMAC on OA treatments [45,46,47,48], research is still debating whether BMAC treatment is equivalent to “stem cell therapy”, such as expanded MSCs, due to the low number of MSCs available in BMAC [49]. We believe that all the growth factors, cytokines, and perhaps even extracellular vesicles or exosomes from MSCs and other progenitors also play very important roles in repairing damaged cartilage and reducing inflammation. Therefore, we were not only focusing on the cells but also on the factors contained in BMAC. We found the freezing process (−80 °C for 4 weeks) for BMAC does not significantly affect the concentration of soluble factors in the plasma from BMAC, except decreasing IL8 and increasing IL15, which are pro-inflammatory factors. Reduced IL-8 may be beneficial, as IL-8 plays a significant role in OA by contributing to inflammation, cartilage degradation, and disease progression [50,51]. In contrast, higher IL-15 expression has been associated with pain and disease severity in OA patients [52,53]. Therefore, while the reduction in IL-8 might attenuate certain inflammatory pathways, the concomitant increase in IL-15 could conversely promote immune-mediated inflammation. These findings suggest that cryopreservation alters the cytokine milieu in a complex manner, with both potentially protective and detrimental effects on OA pathology. Interestingly, the level of IL-10, which is secreted by MSCs and can inhibit the activation of immune cells, thus preventing autoimmune activities and inflammation [54,55], was maintained the same as in fresh BMAC, suggesting that the potential immunomodulatory ability of MSCs was not affected by the freezing process.

Platelets are an important component of BMAC, as they release numerous growth factors, such as PDGF, TGF-β, and VEGF, that contribute to cartilage repair and regeneration [56]. Therefore, reduced platelet viability after freezing may attenuate these paracrine effects and potentially diminish certain therapeutic benefits of BMAC. In this study, we observed a marked decline in platelet counts after cryopreservation. However, it did not affect the cartilage regenerative potential of BMAC in vivo. A decrease in platelet counts may be due to loss, degranulation and decomposition during centrifugation steps and the freezing and thawing process [57,58]. In the future, the maintenance of platelet counts in the BMAC after cryopreservation needs to be further investigated.

The information regarding the biosafety of cryopreserved MSCs is still not well established. Previous studies have shown that cryopreservation processes involving DMSO have the potential to modify the cell cycle and chromosome stability of stem cells and lead to alteration of cell functions that might eventually result in tumorigenesis [59]. In this study, for the frozen BMAC treatment in vivo, we removed DMSO before injection via a wash method using PBS, and by freezing the plasma and cells from BMAC separately to avoid the toxicity of DMSO. This may be one of the methods for clinical use in the future, since the biosafety of other cryoprotectant agents has not yet been well established and the biosafety of cryopreserved MSCs in terms of genetic stability and tumorigenic potential is essential prior to clinical applications. However, total removal of DMSO is complex and time-consuming for clinical use [60]. New research is required to focus on eliminating cryoprotectants during the processing of cellular products and removing these animal components (e.g., Fetal Bovine Serum which is often used in culture media) in the final products prior to their clinical applications in order to prevent the potential to trigger an immune response or transmit pathogens to the recipients [61,62].

In this study, the DMM model was used to induce osteoarthritis. Although this is a well-established model, no differences were observed between the normal and PBS groups in the microCT analysis, suggesting that this model may not have been sensitive enough to detect changes in subchondral bone under our experimental conditions. Therefore, the choice of model may limit the interpretation of the present findings. Although this study has several limitations, i.e., short-term −80 °C storage rather than long-term liquid-nitrogen banking and modest sample size and donor variability with limited stratification, our results demonstrated that short-term freezing of whole BMAC at −80 °C for 4 weeks preserved key MSC functional readouts in vitro and supported cartilage repair in vivo to a degree comparable to fresh BMAC under our conditions. These findings support the feasibility of banking BMAC for staged use, while highlighting platelet and cytokine changes that may require protocol optimization and evaluation under long-term storage conditions. A standardized cryopreservation protocol to effectively preserve MSCs for clinical applications is both essential and challenging. In the future, we will continue to test more BMAC samples from different donors, quantify the number of viable MSCs for in vivo study, increase the freezing time for the in vitro and vivo work, optimize freezing methods, and test more different cryopreservation techniques. It is also important to further investigate the effects of cryopreservation on the related gene expression and differentiation capacity of MSCs preserved in various cryopreservation agents, to provide insight into the molecular changes that may occur following the cell-freezing process.

## 5. Conclusions

In summary, we found that cryopreservation with 10% DMSO and 90% autologous plasma at −80 °C for 4 weeks resulted in decreased platelet counts, with no differences in the multipotent differentiation of MSCs derived from fresh and frozen BMAC. In addition, the promotion of cartilage repair by frozen BMAC was equivalent to that of fresh BMAC. This study provided evidence for the possibility of a single BMAC harvest for multiple injections/treatments at different time points without cell expansion, facilitating cartilage repair and clinical application.

## Figures and Tables

**Table 1:** Primer sequences.

Gene Name	Primer Sequence (5′-3′)	Product Size (bp)
Gapdh	Forward: GTATCGGACGCCTGGTTACC Reverse: ACCAGCTTCCCATTCTCAGC	166
CD90	Forward: CAGCAGTTCACCCATCCAGT Reverse: GATGCCCTCACACTTGACCA	271
Sox2	Forward: GCTACAGCATGATGCAGGACCA Reverse: TCTGCGAGCTGGTCATGGAGTT	134
OCT4	Forward: GCAAAGCAGAAACCCTCGTG Reverse: AACCACACTCGGACCACATC	172
MMP9	Forward: ACAGCGAGACACTAAAGGCC Reverse: GGCAAGTCTTCGGTGTAGCT	139
MMP13	Forward: TCCATCCCGAGACCTCATGT Reverse: CACACGTGGTTCCCTGAGAA	189

## Data Availability

All original data will be made available upon query or request from corresponding authors.
